# Predictive value of radiologic studies for malignant otitis externa: a systematic review and meta-analysis

**DOI:** 10.1016/j.bjorl.2021.08.011

**Published:** 2021-10-26

**Authors:** Do Hyun Kim, Sung Won Kim, Se Hwan Hwang

**Affiliations:** a Department of Otolaryngology-Head and Neck Surgery, Seoul St. Mary’s Hospital, College of Medicine, The Catholic University of Korea, Seoul, South Korea; b Department of Otolaryngology-Head and Neck Surgery, Bucheon St. Mary’s Hospital, College of Medicine, The Catholic University of Korea, Seoul, South Korea

**Keywords:** Otitis externa, Osteomyelitis, Technetium, Gallium-67, Diagnostic imaging

## Abstract

**Objective:**

To determine the diagnostic accuracy of Necrotizing Otitis Externa (NOE) based on radiologic studies.

**Methods:**

The PubMed, Cochrane, Embase, Web of Science, SCOPUS, and Google Scholar databases were searched. True-positive and false-negative results were extracted for each study. Methodological quality was evaluated using the Quality Assessment of Diagnostic Accuracy Studies 2 (QUADAS-2) tool.

**Results:**

The included studies contained data on 37 studies diagnosed with NOE. The sensitivity of gallium-67, technetium-99m, and Magnetic Resonance Imaging (MRI) was 0.9378 (0.7688–0.9856), 0.9699 (0.8839–0.9927), and 0.9417 (0.6968–0.9913), respectively. For Computed Tomography (CT), the positive criteria consisted of bony erosion alone and bony erosion plus any soft tissue abnormality. The sensitivity of CT based only on bony erosion was 0.7062 (0.5954–0.7971); it was higher 0.9572 (0.9000–0.9823) when based on bony erosion plus any soft tissue abnormality.

**Conclusion:**

The diagnostic sensitivity of technetium-99m, gallium-67, and MRI was favorable. On CT, the presence of bony erosion may be a useful diagnostic marker of NOE, but the diagnostic sensitivity will be even higher if the criterion of any soft tissue abnormality is also included; however, care should be taken when interpreting the results. Our study demonstrates the potential utility of radiology studies for diagnosing NOE, but their lack of specificity must be considered, and standardized anatomic criteria are still needed.

**Level of evidence:**

2A.

## Highlights

• The initial diagnosis of necrotizing otitis externa has not been established.
• CT and MRI are the currently preferred imaging modalities.
• The diagnostic sensitivity of technetium-99m, gallium-67, and MRI was favorable.

## Introduction

Necrotizing Otitis Externa (NOE), also referred to as malignant external otitis, malignant otitis externa, invasive otitis externa, and skull base osteomyelitis, is an invasive bacterial infection of the external ear canal and skull base that frequently results in bone erosion. It is a rare but life-threatening complication of external otitis that often develops in diabetic and immunocompromised patients.^1^ In 1987, Cohen and Friedman described diagnostic criteria for NOE. Major criteria include symptoms and signs of otalgia, otorrhea, edema, granulation tissue, and postoperative micro abscess, as well as imaging findings including a positive bone scan with technetium-99m or gallium-67. Positive findings on Computed Tomography (CT) were classified as a minor criterion.^2^, ^3^

There is still a lack of consensus regarding the imaging modality most suitable for the initial diagnosis of NOE. A large cross-sectional study by the American Neurotology Society and The American Society of Head and Neck Radiology found considerable heterogeneity in the preferred imaging modalities for the initial diagnosis of NOE.^4^ In a recent survey, clinicians preferred CT and Magnetic Resonance Imaging (MRI) to nuclear medicine imaging for diagnosing NOE.^4^ However, most evidence-based knowledge has been derived from small case series or cohort studies, such that the diagnostic accuracy of imaging studies for NOE is unclear.^5^ We therefore performed a meta-analysis to determine the diagnostic accuracy of radiologic studies for NOE.

## Methods

### Ethical considerations

This review study did not treat human participants. Therefore, our Institutional Review Board waived the need for informed consent for this systematic review and meta-analysis.

### Literature search

Clinical studies were retrieved from PubMed, the Cochrane Central Register of Controlled Trials, Embase, Web of Science, SCOPUS, and Google Scholar; the search period was the date of each database’s inception to February 2021. The search terms were as follows: “necrotizing otitis externa”, “malignant otitis externa”, “skull base osteomyelitis”, “imaging study”, “radiology”, “nuclear medicine imaging”, “technetium”, “gallium”, “MRI”, and “CT”. Only studies written in English were reviewed. The reference lists were examined to ensure that no relevant studies were omitted. All abstracts, as well as the titles of candidate studies, were reviewed by two independent reviewers.

### Selection criteria

**Figure 1:**
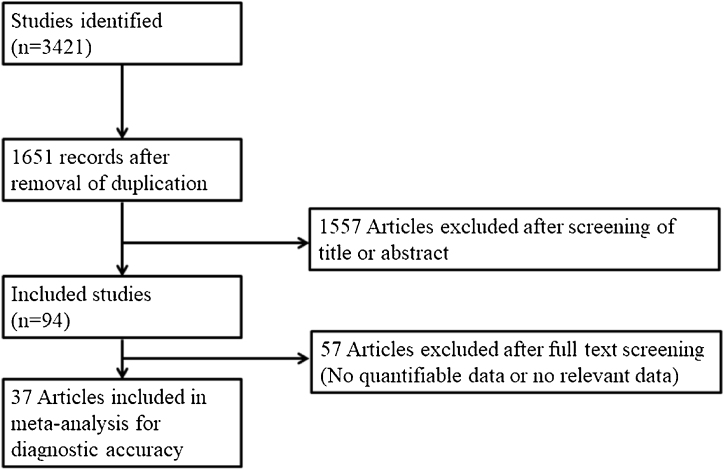
Summary of the literature search strategy.

The inclusion criteria were: (1) Patients diagnosed with NOE; (2) A prospective or retrospective study protocol; (3) >10 cases; and (4) Sensitivity analysis of radiologic studies. The exclusion criteria were: (1) Case report format; (2) Review article; and (3) A lack of diagnostic radiologic study data. The search strategy is summarized in Fig. 1. Participants, Interventions, Comparisons, Outcomes, Timings, and Study design (PICOTS) were summarized in the Supplementary Table S1.

### Data extraction and risk of bias assessment

All data were collected using a standardized form.^6^ Since the included studies only enrolled patients diagnosed with NOE, there were no false-positives or true-negatives. Therefore, the specificity, diagnostic odds ratio, and area under the summary receiver operating characteristic curve could not be assessed. The sensitivity values obtained from diagnostic technetium-99m,^7^, ^8^, ^9^, ^10^, ^11^, ^12^, ^13^, ^14^, ^15^, ^16^, ^17^, ^18^, ^19^, ^20^, ^21^, ^22^, ^23^, ^24^, ^25^, ^26^, ^27^, ^28^ gallium-67,^9^, ^10^, ^17^, ^22^^,^^25^, ^27^, ^28^, ^29^, ^30^, ^31^ MRI,^24^, ^26^, ^28^, ^32^, ^33^, ^34^ and CT^1^, ^7^, ^15^, ^16^, ^17^, ^18^, ^19^, ^20^, ^23^, ^24^, ^25^, ^26^, ^27^, ^28^, ^30^, ^32^, ^33^, ^34^, ^35^, ^36^, ^37^, ^38^, ^39^, ^40^, ^41^, ^42^ assessments were analyzed. The quality of the studies was analyzed using the Quality Assessment of Diagnostic Accuracy Studies 2 (QUADAS-2) tool.^43^

### Statistical analysis and outcome measurements

The meta-analysis was carried out using R statistical software (R Foundation for Statistical Computing, Vienna, Austria). Homogeneity was analyzed using the Q statistic and forest plots of sensitivity were drawn. In the sensitivity analysis, studies were excluded one at a time to determine their influence on the overall effect size.

## Results

Thirty-seven studies were included in this analysis. Their characteristics are listed in Supplementary Table S2 and the bias assessment is shown in Supplementary Table S3.

### Nuclear medicine imaging

**Figure 2:**
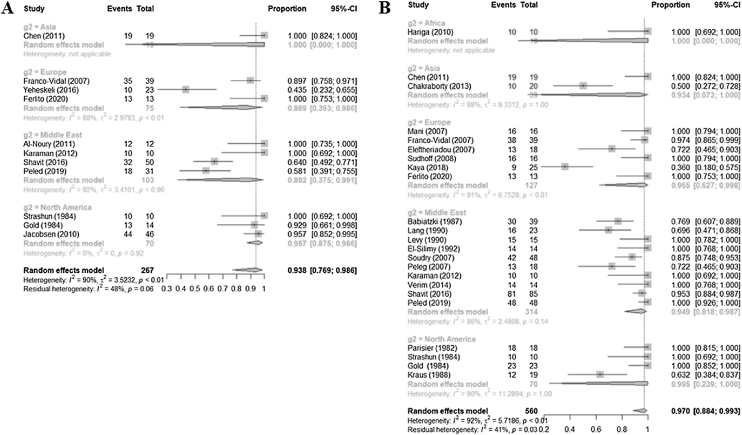
Forest plot of the sensitivity of technetium-99m (A) and gallium-67 (B) scans.

For the 23 retrospective studies based on technetium-99m scans, the sensitivity for diagnosing NOE was 0.9699 (0.8839–0.9927, I^2^ = 92.3%), and for the 10 retrospective studies based on gallium-67 it was 0.9378 (0.7688–0.9856, I^2^ = 90.3%) (Fig. 2).

Subgroup analyses according to the location of the medical center were performed given the high heterogeneity in the sensitivity values (Table 1). The sensitivity of technetium-99m studies in the North American subgroup (0.995) was higher than that of the Middle Eastern (0.949), European (0.965), and Asian (0.933) subgroups (*p* = 0.9680). Similarly, the sensitivity of gallium-67 studies in the North American subgroup (0.957) was higher than that of the Middle Eastern (0.892) and European (0.869) subgroups (Table 1). However, the differences were not statistically significant. Overall, the results showed that nuclear medicine imaging is diagnostically powerful regardless of where it is performed.

**Table 1:** Diagnostic value of nuclear medicine studies according to the locations of the medical centers included in the meta-analysis.

Subgroup analysis	Study (n)	Sensitivity (95% CIs); I^2^	Subgroup analysis	Study (n)	Sensitivity (95% CIs); I^2^
Technetium-99m	23	0.9699 (0.8839–0.9927); 92.3%	Gallium-67	11	0.9378 (0.7688–0.9856); 90.3%
Africa	1	1.0000 (0.0000–1.0000); NA	Asia	1	1.0000 (0.0000–1.0000); NA
Asia	2	0.9336 (0.0734–0.9996); 88.1%	Europe	3	0.8692 (0.3934–0.9855); 88.3%
Europe	6	0.9654 (0.6266; 0.9979); 91.3%	Middle East	4	0.8921 (0.3748–0.9913); 92.3%
Middle East	9	0.9494 (0.8185–0.9874); 85.9%	North America	3	0.9571 (0.8754–0.9861); 0%
North America	4	0.9951 (0.2391–1.0000); 90.1%			

NA, not applicable.

### CT and MRI

**Figure 3:**
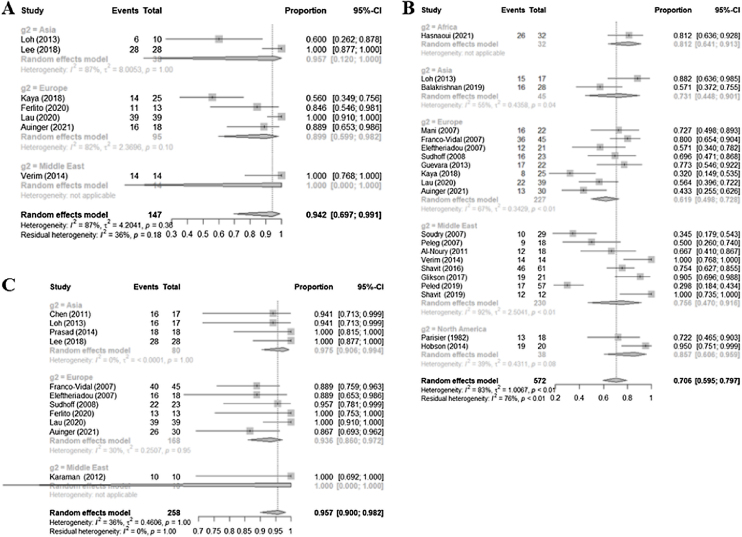
Forest plot of the sensitivity of MRI (A), CT with only bony erosion (B), and CT with bony erosion plus any abnormality of the soft tissue (C).

The sensitivity of MRI based on the seven retrospective studies included in the meta-analysis was 0.9417 (0.6968–0.9913; I^2^ = 87.2%). For CT, the positive criteria defined in the studies were bony erosion (n = 21) and bony erosion plus any soft tissue abnormality (n = 11). The sensitivity of CT using bony erosion as the sole criterion was 0.7062 (0.5954–0.7971; I^2^ = 83.4%) the sensitivity was higher for bony erosion plus any soft tissue abnormality, at 0.9572 (0.9000–0.9823, I^2^ = 36.3%) (Fig. 3).

Again, subgroup analyses were performed based on the location of the medical center, given the high heterogeneity of the sensitivity values (Table 2). For MRI, the Middle Eastern subgroup had the highest sensitivity, but this subgroup comprised only one study. The sensitivities of the Asian and European subgroups were 0.9566 and 0.899, respectively. For CT based solely on the finding of bony erosion, the sensitivity of the North American subgroup was higher (0.857) than that of the Middle Eastern (0.756), European (0.619), and Asian (0.731) subgroups, although the difference was not statistically significant (*p* = 0.1999).

**Table 2:** Diagnostic value of CT and MRI according to the locations of the medical centers included in the meta-analysis.

Subgroup analysis	Study (n)	Sensitivity (95% CIs); I^2^	Subgroup analysis	Study (n)	Sensitivity (95% CIs); I^2^
MRI	6	0.9655 (0.7207–0.9967); 86.9%	CT (only bony erosion)	21	0.7062 (0.5954–0.7971); 83.4%
			Africa	1	0.8125 (0.6408; 0.9133)
Asia	1	1.0000 (0.0000–1.0000); NA	Asia	2	0.7313 (0.4475–0.9015); 55.0%
Europe	4	0.8994 (0.5991–0.9817); 82.0%	Europe	8	0.6194 (0.4975–0.7278); 66.9%
Middle East	1	1.0000 (0.0000–1.0000); NA	Middle East	8	0.7565 (0.4703–0.9157); 92.1%
			North America	2	0.8569 (0.6057–0.9589); 39.3%

NA, not applicable.

## Discussion

To the best of our knowledge, this is the largest meta-analysis of the diagnostic accuracy of different radiologic studies for NOE. In our analysis of 37 studies, the pooled sensitivity of technetium-99 m was 0.9699 and that of gallium-67 was 0.9378. Although these values are suboptimal, given that NOE is a life-threatening disease with high morbidity, both modalities have sufficiently diagnostic reliability. In previous studies, the high sensitivity of nuclear medicine studies was attributed to the relatively high concentration of radiotracer in areas of increased osteoblastic activity, such as in sites of infection, trauma, and neoplasm.^23^ Although NOE is a severe and progressive infection, it is also rare, and the diagnostic accuracy of the imaging modalities could not be compared in NOE patients vs. a control group. Consequently, neither the true-negative nor false-positive rates of the various imaging modalities, nor the specificity of nuclear medicine studies, could be determined. Also, the modalities can yield positive results for malignant tumors or trauma,^40^ which limits their specificity. Nevertheless, diagnostic imaging for NOE remains useful given the high sensitivity of the examined modalities.^5^

Unlike in our study, Moss et al. argued against using technetium-99m and gallium-67 studies for the diagnosis of NOE.^3^ The authors reported pooled sensitivities for technetium-99m and gallium-67 of 85% (95% CI 72%–98.1%) and 71.2% (95% CI 55.1%–87.3%), respectively. However, although more than half of the studies included by Moss et al. had a sensitivity of 100% in the forest plot, the authors did not speculate as to the reason for either the unexpectedly low pooled sensitivity or the heterogeneity in sensitivity (36%–100%). The discrepancy between our meta-analysis and that of Moss et al. may have been due to the fact that we included four additional studies, Moreover, Moss et al. used the Excel spreadsheet of Jeruza Lavanholi Neyeloff^44^ rather than a statistical package such as STATA or R.^45^

With increasing acceptance of CT and MRI as the preferred diagnostic modalities for NOE, the use of nuclear medicine scans has declined.^3^ An advantage of CT and MRI is that they can identify other diseases besides NOE. However, there have been no published studies demonstrating the diagnostic accuracy of these imaging modalities. We calculated pooled sensitivities for MRI and CT of 0.7062 and 0.9417, respectively, based only on bony erosion. Although MRI is generally less useful for revealing bone involvement, it has high sensitivity for soft tissue disease and may thus reveal early medullary bone and dural involvement.^4^ This advantage is similar to that of nuclear medicine with respect to the detection of early stage osteitis.^23^, ^46^ Although CT is sensitive to bone erosion, radiologic changes are evident only when at least one-third of the bone mineral is lost,^23^ which would explain the difference in sensitivity between MRI and CT when only bony erosion is considered.

For bony erosion together with any soft tissue abnormality, CT had a diagnostic sensitivity of 0.9572 for NOE. Many of the studies evaluated in our meta-analysis reported an increased soft tissue density of the external auditory canal or surrounding tissue on CT. However, this finding is also seen in otitis externa and thus does not confirm NOE, a disadvantage that also characterizes nuclear medicine studies.^33^ In particular, as the specificity of this finding for NOE has not been critically evaluated, CT abnormalities should be interpreted with caution. However, the exact anatomical location of the disease can be accurately evaluated by CT or MRI, along with disease extension.^20^ With further technological advances in both CT and MRI,^4^ they may eventually also be of value in cases of suspected NOE. Also, since CT and MRI have their own advantages and are complementary to each other, it is expected that the diagnosis accuracy of NOE will be higher if they are simultaneously performed.

This analysis had several limitations. First, it included only six MRI studies (one from Asia, one from the Middle East and four from Europe), and many geographic regions were not represented in terms of the clinical presentation and characteristics of NOE. More studies need to be conducted to confirm the high diagnostic power of MRI. Second, the specificity of the imaging modalities was not evaluated in any of the included studies, given the rarity of NOE. Thus, while CT and MRI were shown to be highly sensitive, further clinical studies are needed to rule out false-positives.^3^ Third, there was high heterogeneity in the diagnostic sensitivity of the different radiologic modalities for NOE. This may have been because the studies were conducted at different institutions over several decades, such that there were differences in technology and diagnostic criteria. Standardized data collection and a larger number of cases are required to address these issues.

## Conclusion

The results of this systematic review and meta-analysis confirmed the high sensitivity of technetium-99 and gallium-67 for diagnosing NOE, consistent with the data for both methods that have accrued since the 1980s. However, CT and MRI are the currently preferred imaging modalities, as both are able to reveal the precise anatomical location of the disease and their diagnostic sensitivity is sufficiently high. However, their specificity for diagnosing NOE remains to be determined. Thus, CT or MRI studies to detect NOE must be accompanied by a careful clinical evaluation.

## Conflicts of interest

The authors declare no conflicts of interest.
