# Supplementary material for: Predictive value of radiologic studies for malignant otitis externa: a systematic review and meta-analysis
Source: Braz J Otorhinolaryngol. 2021 Oct 26;89(1):66–72. doi: 10.1016/j.bjorl.2021.08.011 (PMC9874358; doi:10.1016/j.bjorl.2021.08.011)
Supplement: Supplementary file 1 [file mmc1.docx]

JPED-D-21-00484 – Supplementary Material

**Supplementary Table 1** Participants, Interventions, Comparisons, Outcomes, Timings, and Study design (PICOTS).

| **Participants** | Patients diagnosed with necrotizing otitis externa |
| --- | --- |
| **Interventions** | Diagnostic radiologic study: technetium-99m or gallium-67 nuclear medicine imaging, computed tomography, or magnetic resonance imaging |
| **Comparisons** | Comparison between each group |
| **Outcomes** | Diagnostic accuracy, sensitivity analysis |
| **Timings** | From inception to February 2021 |
| **Study design** | A systematic review and meta-analysis |

**Supplementary Table 2** Study characteristics.

| **Study** | **Number** | **Age, median (range) or mean (SD), y** | **Sex (male/ female)** | **Location** | **Comorbidity** | **Complete remission** | **Measurement** |
| --- | --- | --- | --- | --- | --- | --- | --- |
|  |  |  |  |  |  |  |  |
| Parisier (1982) | 18 | 75 (61–89) | 13/5 | North America | Diabetes (100%) | Death rate = 3/18 | Tc, CT |
| Gold (1984) | 23 | 60–89 | 18/5 | North America | Diabetes (100%) | Death rate = 3/23 | Tc, Ga |
| Strashun (1984) | 10 | 68 (61–86) | 8/2 | North America | Diabetes (90%)/ non-Diabetes (10%) | Complete remission = 7/10 | Tc, Ga |
| Babiatzki (1987) | 50 | 67.2 (32–92) | 36/14 | Middle East | Diabetes (68%)/ non-Diabetes (32%) | Overall mortality rate = 10% | Tc |
| Kraus (1988) | 19 | 70 (49–90) | 18/1 | North America | Diabetes (84%)/ immunosuppression (16%) | Death rate = 3/19 | Tc |
| Lang (1990) | 23 | 67 (54–92) | 16/7 | Middle East | NR | Treatment failure = 2/23 | Tc |
| Levy (1990) | 17 | 69 (48–85) | 11/6 | Middle East | Diabetes (41%)/ non-Diabetes (59%) | Complete remission = 17/17 | Tc |
| El-Silimy (1992) | 14 | 56.8 (2–83) | 8/4 | Middle East | Diabetes (95%)/ immunosuppression (5%) | Complete remission = 14/14 | Tc |
| Eleftheriadou (2007) | 21 | 63 (40–88) | 12/9 | Europe | Diabetes (95%)/ immunosuppression (5%) | NR | Tc, CT |
| Franco-Vidal (2007) | 46 | 73.6 (23–91) | 32/14 | Europe | Diabetes (65.2%)/ immunosuppression (17.4%) | Overall cure rate = 95.6% | Tc, Ga, CT |
| Mani (2007) | 23 | 71 (39–87) | 19/4 | Europe | Diabetes (91%)/ non-Diabetes (9%) | Overall cure rate = 87% | Tc, CT |
| Peleg (2007) | 18 | NR | NR | Middle East | Diabetes (94%)/ non-Diabetes (6%) | NR | Tc, CT |
| Soudry (2007) | 48 | 78 | 31/17 | Middle East | Diabetes (91%)/ non-Diabetes (9%) | Death rate = 19/48 | Tc, CT |
| Sudhoff (2008) | 23 | 71 (39–87) | 19/4 | Europe | Diabetes (94.7%)/ non-Diabetes (5.3%) | Complete remission = 23/23 | Tc, CT |
| Hariga (2010) | 19 | 72 (23–84) | 10/9 | Africa | Diabetes (94.7%)/ non-Diabetes (5.3%) | Overall cure rate = 89.4% | Tc |
| Jacobsen (2010) | 46 | 62 (22–89) | NR | North America | Diabetes (80%)/ non-Diabetes (20%) | Overall cure rate = 72% | Ga |
| Al-Noury (2011) | 18 | 65.3 (49–79) | 16/2 | Middle East | Diabetes (100%) | Death rate = 2/18 | Ga, CT |
| Chen (2011) | 19 | 67.3 (38–83) | 12/7 | Asia | Diabetes (73%)/ non-Diabetes (17%) | Death rate = 2/19 | Tc, Ga, CT |
| Karaman (2012) | 10 | 70 (64–82) | 7/3 | Middle East | Diabetes (90%)/ non-Diabetes (10%) | Complete remission = 10/10 | Tc, Ga, CT |
| Guevara (2013) | 22 | 70 | 17/5 | Europe | Diabetes (55%)/ non-Diabetes (45%) | NR | CT |
| Loh (2013) | 19 | 69.1 (51–86) | 16/3 | Asia | Diabetes (94%)/ non-Diabetes (6%) (all immunocompromized) | Complete remission = 16/19 | CT, MRI |
| Chakraborty (2013) | 20 | 62 (48–79) | 14/6 | Asia | Diabetes (100%) | NR | Tc |
| Hobson (2014) | 20 | 65 | NR | North America | Diabetes (75%)/ non-Diabetes (25%) | Complete remission = 15/20 | CT |
| Prasad (2014) | 18 | 50 (40~50) | 9/9 | Asia | NR | Complete remission = 18/18 | CT |
| Verim (2014) | 14 | 69.57 (58–79) | NR | Middle East | Diabetes (100%) | Death rate = 0/14 | Tc, CT, MRI |
| Shavit (2016) | 88 | 73±11.5 | 61/27 | Middle East | Diabetes (75%)/ non-Diabetes (25%) | Disease specific mortality 14% | Tc, Ga, CT |
| Yeheskeli (2016) | 23 | 73.2 (40–86) | 15/8 | Europe | Diabetes (64%)/ non-Diabetes (36%) | Death rate = 4/23 | Ga |
| Glikson (2017) | 25 | 73.8 (27–93) | 18/7 | Middle East | Diabetes (84%)/ immunosuppression (8%) | Death rate = 2/25 | CT |
| Kaya (2018) | 25 | 69.68±11.29 | 18/7 | Europe | Diabetes (100%) | NR | Tc, CT, MRI |
| Lee (2018) | 28 | 65 (33–81) | 22/6 | Asia | Diabetes (82%)/ non-Diabetes (18%) | Death rate = 4/28 | CT, MRI |
| Balakrishnan (2019) | 28 | 65.36 (45–83) | 24/4 | Asia | Diabetes (89%)/ non-Diabetes (11%) | Death rate = 4/28 | CT |
| Peled (2019) | 81 | 68.2 | 48/33 | Middle East | Diabetes (92.5%)/ non-Diabetes (7.5%) | Death rate = 2/81 | Tc, Ga, CT |
| Shavit (2019) | 12 | 74±11.5 | 10/2 | Middle East | Diabetes (83%)/ immunosuppression (4%) | Death rate = 2/12 | CT |
| Lau (2020) | 39 | 74 (43–92) | 13/26 | Europe | Diabeticor immunocompromised (79.5%) | NR | CT, MRI |
| Ferlito (2020) | 13 | 70.6 (54–82) | 10/3 | Europe | Diabetes (69%)/ immunosuppression (31%) | Complete remission = 13/13 | Tc, Ga, CT, MRI |
| Hasnaoui (2021) | 40 | 65±12.9 | 19/21 | Africa | Diabetes (90%)/ Unknown (10%) | Complete remission = 40/40 | CT |
| Auinger (2021) | 30 | 73.1±13.5 | 20/10 | Europe | Diabetes (73.3%)/ immunosuppression (26.7%) | Death rate = 11/30 | CT, MRI |

NR, Not Reported; Tc, Technetium-99m; CT, Computed Tomography; Ga, Gallium scan; MRI, Magnetic Resonance Imaging.

**Supplementary Table 3** Bias assessment.

| **Reference** | **Risk of Bias** | | | | **Concerns about application** | | |
| --- | --- | --- | --- | --- | --- | --- | --- |
|  | **Patient selection** | **Index test** | **Reference standard** | **Flow and timing** | **Patient selection** | **Index test** | **Reference standard** |
| Parisier (1982) | Low | Low | Unclear | Low | Low | Low | Low |
| Gold (1984) | Unclear | Low | Low | Low | Low | Low | Low |
| Strashun (1984) | Unclear | Low | Low | Low | Low | Low | Low |
| Babiatzki (1987) | Low | Low | Unclear | Low | Low | Low | Low |
| Kraus (1988) | Low | Low | Low | Low | Low | Low | Low |
| Lang (1990) | Low | Low | Unclear | Low | Low | Low | Low |
| Levy (1990) | Unclear | Low | Low | Low | Low | Low | Low |
| El-Silimy (1992) | Low | Low | Unclear | Low | Low | Low | Low |
| Eleftheriadou (2007) | Low | Low | Unclear | Low | Low | Low | Low |
| Franco-Vidal (2007) | Unclear | Low | Low | Low | Low | Low | Low |
| Mani (2007) | Low | Low | Low | Low | Low | Low | Low |
| Peleg (2007) | Low | Low | Unclear | Low | Low | Low | Low |
| Soudry (2007) | Unclear | Low | Low | Low | Low | Low | Low |
| Sudhoff (2008 | Low | Low | Unclear | Low | Low | Low | Low |
| Hariga (2010) | Low | Low | Unclear | Low | Low | Low | Low |
| Jacobsen (2010) | Low | Low | Low | Low | Low | Low | Low |
| Al-Noury (2011 | Low | Low | Low | Low | Low | Low | Low |
| Chen (2011) | Unclear | Low | Low | Low | Low | Low | Low |
| Karaman (2012) | Unclear | Low | Low | Low | Low | Low | Low |
| Guevara (2013) | Low | Low | Unclear | High | Low | Low | Low |
| Loh (2013) | Low | Low | Low | Low | Low | Low | Low |
| Chakraborty (2013) | Low | Low | Unclear | Low | Low | Low | Low |
| Hobson (2014) | Unclear | Low | Low | Low | Low | Low | Low |
| Prasad (2014) | Low | Low | Unclear | Low | Low | Low | Low |
| Verim (2014) | Low | Low | Low | Low | Low | Low | Low |
| Shavit (2016) | Unclear | Low | Low | Low | Low | Low | Low |
| Yeheskeli (2016) | Low | Low | Low | Low | Low | Low | Low |
| Glikson (2017) | Low | Low | Unclear | Low | Low | Low | Low |
| Kaya (2018) | Unclear | Low | Low | Low | Low | Low | Low |
| Lee (2018) | Low | Low | Low | Low | Low | Low | Low |
| Balakrishnan (2019) | Low | Low | Unclear | Low | Low | Low | Low |
| Peled (2019) | Unclear | Low | Low | Low | Low | Low | Low |
| Shavit (2019) | Low | Low | Low | Low | Low | Low | Low |
| Lau (2020) | Low | Low | Low | Low | Low | Low | Low |
| Hasnaoui (2021) | Unclear | Low | Low | Low | Low | Low | Low |
| Auinger (2021) | Low | Low | Low | Low | Low | Low | Low |
| Ferlito (2020) | Low | Low | Low | Low | Low | Low | Low |
